# A comprehensive approach to women’s health: lessons from the Mexican health reform

**DOI:** 10.1186/1472-6874-12-42

**Published:** 2012-12-10

**Authors:** Julio Frenk, Octavio Gómez-Dantés, Ana Langer

**Affiliations:** 1 Harvard School of Public Health, 677 Huntington Avenue, Boston, MA, 02115, USA; 2 Center for Health Systems Research, National Institute of Public Health, Avenida Universidad 655, C.P. 62100, Cuernavaca, Morelos, Mexico; 3 Women and Health Initiative, Harvard School of Public Health, 677 Huntington Avenue, Boston, MA, 02115, USA

**Keywords:** Women and health, Mexican health reform, Fair Start in Life

## Abstract

**Background:**

This paper discusses the way in which women’s health concerns were addressed in Mexico as part of a health system reform.

**Discussion:**

The first part sets the context by examining the growing complexity that characterizes the global health field, where women’s needs occupy center stage. Part two briefly describes a critical conceptual evolution, *i.e.* from maternal to reproductive to women’s health. In the third and last section, the novel “women *and* health” (W&H) approach and its translation into policies and programs in the context of a structural health reform in Mexico is discussed. W&H simultaneously focuses on women’s health needs and women’s critical roles as both formal and informal providers of health care, and the links between these two dimensions.

**Summary:**

The most important message of this paper is that broad changes in health systems offer the opportunity to address women’s health needs through innovative approaches focused on promoting gender equality and empowering women as drivers of change.

## Background

Despite important global progress, women’s health and, in particular, sexual and reproductive health, are still very much a part of the unfinished agenda. Indeed, girls and women face health challenges that have not yet been fully addressed. In fact, millions of women lack access to basic life-saving services and hundreds of thousands of women suffer death or disability every year from preventable diseases and complications of pregnancy. Every two minutes a woman unnecessarily dies of preventable pregnancy-related complications, [1] leaving behind impoverished orphans, struggling families, and devastated communities. Around the world, 200 million adolescent girls and women do not have access to safe and effective contraception —the fundamental tool for controlling their reproductive lives. Females still struggle with unwanted pregnancy, maternal morbidity and mortality, unsafe abortion, and reproductive cancers.

Along with the biological risks, women and girls are affected by gender and other social inequalities, which are the underlying conditions for pervasive problems such as gender-based violence and the “feminization” of the HIV/AIDS epidemic. At later stages of life, women receive poorer and later care for problems they share with men, such as cardiovascular disease and lung cancer.

Novel visions and strategies are required to tackle girls’ and women’s unmet health needs and to challenge gender and other social inequalities. Efforts to address each of these issues must recognize that women are, simultaneously, consumers and producers of health care [2]. Mothers, sisters, and grandmothers are the primary caretakers in their households, and female community workers and midwives serve people who otherwise lack access to health services because they live in remote areas or cannot afford care. Women often are the most active members of community health committees and other forms of organization of civil society. They also represent a majority in the health professions, make key contributions to the health sciences and academia, and strategically set priorities and allocate health care funding as decision makers around the globe. Gender-related barriers and lack of enabling policies to help balance life and work prevent women from achieving their full potential in the health workforce.

The purposes of this paper are to present a concrete experience that illustrates this comprehensive approach and to discuss its translation into policies and programs. We analyze this experience in the context of a structural health reform implemented in Mexico between 2000 and 2006 and the growing complexity that characterizes the global health field, where women’s needs occupy center stage. The framework for our discussion is a critical conceptual evolution that has taken place in the last decades, *i.e.* from maternal to reproductive to women’s health throughout the life cycle. The most important message we want to convey through this paper is that broad changes in health systems offer the opportunity to address women’s health needs through innovative approaches focused on promoting gender equality and empowering women as drivers of change.

## Discussion

### Challenges of global health

Today we are all keenly aware that in health matters the world has become a neighborhood: “a place you are already in when you walk out your door [3]”. This awareness comes at a time of unprecedented change: we are in the midst of a major health transition unlike anything the world has seen before.

To begin with, during the 20^th^ century the world as a whole experienced a larger gain in life expectancy than in all the previously accumulated history of humankind. Average life expectancy in the world increased from 30 years in 1900 to 66.6 years in 2009.

We have also witnessed a shift in the dominant patterns of disease. The relative importance of different causes of death has changed from acute infections in children to chronic non-communicable disorders in adults.

The whole meaning of disease has also been transformed. Previously, the experience of disease was marked by a succession of acute episodes from which one either recovered or died. Now people spend substantial parts of their lives in less than perfect health, coping with a chronic condition, often stigmatized.

The ongoing health revolution has undoubtedly produced enormous benefits, but it has also opened new challenges. Equity is the most daunting of all. Progress on the health transition has not been shared equally by all nations of the world. Whereas rich countries experienced a substitution of old for new patterns of disease, the developing world is simultaneously facing a triple burden of ill health: first, the unfinished agenda of infections, under-nutrition, and reproductive health problems; second, the emerging challenges represented by non-communicable diseases, mental disorders, and the growing scourge of injury and violence; and third, the health risks associated with globalization, including the threat of pandemics like AIDS and influenza, the trade in harmful products like tobacco and other drugs, the health consequences of climate change, and the dissemination of harmful lifestyles leading to the epidemic of obesity.

### From maternal to reproductive to Women’s health

In the context of this protracted health transition, the concepts that reflect the priority of different aspects of women’s health have themselves been evolving. Until late last century, the concept that prevailed was that of ‘maternal and child health’ (MCH), which considered the well-being of women as a vehicle to improve childrenÂ´s health, instead of a legitimate end in itself. In the mid-1980s, this insufficient attention to women was strongly confronted by Rosenfield and Maine in their classical article on the invisibility of the maternal health component in the maternal and child health programs [4]. A few years later, UN bodies and non-governmental organizations launched the Safe Motherhood Initiative (SMI), which represented the first global effort to address maternal health. Focused on health during pregnancy, delivery and the post-partum, the SMI meant a gradual shift from an exclusive concern for survival to a broader interest in the prevention of disability and the positive promotion of well-being [5].

It is important to mention that a work written by the Boston Women’s Health Book Collective in the early 1970s, *Our Bodies, Ourselves*, had disseminated a broader approach to women’s health that was well ahead of its time. This book contained information on many issues related to women’s health and sexuality, including birth control, childbirth, sexual health, sexual orientation, and gender identity [6].

While maternal health was slowly gaining visibility, in the mid 1990’s the international health community, prompted by a solid grassroots movement, moved beyond a demographic/population control approach and embraced the comprehensive concept of sexual and reproductive health and rights (SRHR) that includes maternal health, but transcends it [7]. In 1994 and 1995, at the International Conference on Population and Development in Cairo and the Fourth World Conference for Women in Beijing, respectively, the vast majority of countries officially adopted the new SRHR paradigm [8,9].

Over the following years, attention on maternal health gradually increased and safe and effective interventions that allow preventing maternal deaths when accessible to women with obstetric complications were developed [10]. In spite of this progress, maternal health continues to be a major priority in the global health agenda: approximately 300 thousand women every year are still losing their lives in the process of giving life [1,11]. The fact that 99% of these deaths happen in developing countries makes maternal mortality ratio the most inequitably distributed health indicator in the world [12]. The inacceptable persistence of maternal mortality and its association with poverty prompted the global community to dedicate one of the eight Millennium Development Goals (MDG number 5) to its reduction, committing to bring the 1990 levels down by two-thirds in 2015 [13].

In more recent years, to a great extent because of a rising awareness of the inextricable links between women’s health conditions along the life cycle and the increasing prevalence of non-communicable diseases among women in developing countries, the expanded concept of ‘women’s health’ gained currency in the global arena. “Women’s health” has maternal and reproductive health at its core, but goes beyond that to include emerging challenges that are both practically exclusive to women—like cervical and breast cancer—or particularly common among them, such as depression, the increasing consumption of tobacco, and obesity [14]. This new emphasis adds an additional layer of complexity to the health challenges women face. In the last few years, other broader approaches to women’s health have been promoted, including the economic dimension of maternal health and “women-centered development” [15,16].

In Mexico, a comprehensive approach to women’s health was developed around the concept of ‘women and health’ and implemented between 2000 and 2006. This effort was part of a larger initiative to reform the health system [17-23]. The main features of the reform are briefly described below as the larger context for the implementation of the broad and ambitious women and health agenda.

### Addressing Women’s concerns in the context of the Mexican health reform

The calculation of national health accounts in Mexico in the 1990s revealed that more than 50% of total health expenditure was out-of-pocket, due the fact that more than half of the population (around 50 million people) lacked health insurance [24]. This type of expenditures exposed households to ruinous situations. In fact, further analyses demonstrated that close to 4 million households were paying catastrophic and/or impoverishing sums to meet the health needs of their family members [25].

These and other analyses generated the evidence that supported a legislative reform that established a system of social protection in health, which was approved by a large majority of the Mexican Congress in 2003. One of the main objectives of this reform was to increase public funding by a full percentage point of the GDP over seven years in order to provide universal health insurance. The vehicle for achieving this aim is a public scheme called *Seguro Popular* (Popular Health Insurance in English), which guarantees regular access to a package of more than 250 essential interventions which include all services offered in ambulatory units and general hospitals of the Ministry of Health (MoH), and a package of 57 costly interventions which includes treatment for all types of cancer in children, cervical and breast cancer, and HIV/AIDS [26]. This insurance has elicited an enthusiastic response from the population, so that by December of 2011 more than 51.8 million people were enrolled in it and the target of universal coverage has been reached in 2012 [27,28].

In the context of a structural reform addressing the cross-cutting challenge of financing universal access to high-quality services, it was necessary to have a clear sense of priorities. This was seen as an imperative not only in terms of resource allocation, but also to garner public support by relating the abstract financial and managerial notions to concrete deliverables. Every reform must have a limited number of “flagship initiatives” to focus attention on its concrete benefits. From the outset, it was decided that a comprehensive approach to address women’s health’ would be one of them.

The first and most pressing priority in Mexico was to reduce maternal deaths. Having already achieved over 95% coverage with one of the most complete immunization schedules in the world [29], the next frontier for equity was to close the social gaps in maternal mortality. Indeed, at the beginning of this decade, the progress reports produced by the United Nations showed that Mexico was one of the very few developing countries that were on route to meet the health-related MDGs except for one indicator: maternal mortality. In 1990 the maternal mortality ratio for Mexico was 90.4 and in order to meet MDG 5 it should decline to 22.6 in 2015, a goal that looks unreachable, given the present level of this indicator (36.1 in 2010) [30]. Like for the rest of the world, this was the indicator exhibiting the highest degree of inequality across social groups and regions of the country. Even though maternal deaths on average had decreased consistently in the previous decade, major efforts were still needed in the poorest areas of the southern states of Mexico, where geographic, organizational, financial, and cultural barriers limited women’s access to maternal health care [31]. The differences among states were huge. The northern state of Nuevo León had a maternal mortality ratio of 16 in 2004 while the southern state of Chiapas showed a ratio of 103 [32].

In rural areas of Mexico maternal deaths were mostly associated to acute obstetric hemorrhage, which demanded improvements in timely access to skilled delivery care. Hemorrhage was responsible for 25% of maternal deaths in 2004 [29]. In urban settings most maternal deaths were due to eclampsia, which concentrated 30% of maternal deaths nationally that same year. Indirect obstetric ailments were the main cause of maternal deaths.

To address maternal morbidity and mortality and closely related perinatal health challenges, the special “Fair Start in Life” initiative (“Arranque Parejo en la Vida” in Spanish) was launched in 2001. The main purposes of the initiative were to address the health problems of newborns and children under 5 and the reduction of maternal mortality. The name of program was meant to underscore the fundamental value of equality of opportunity.

The maternal component of the program included specific budget allocations to strengthen health-care networks and the supply of drugs and other inputs, including safe blood. The availability of skilled human resources was also improved through the reintroduction of obstetric nurses, a figure that began to disappear in the last quarter of the past century as pregnancy and delivery became increasingly medicalized [33]. The training of traditional birth attendants by NGOs and *ad hoc* groups organized by local health authorities was also strengthened to respond to the demands of women in indigenous communities. Measures were taken to expand coverage of antenatal care and institutional deliveries, with emphasis on timely diagnosis and treatment of obstetric emergencies. Finally, efforts were developed to monitor maternal deaths, including the review of all deaths of women of reproductive age through verbal autopsies [34].

As a result of these measures, effective coverage of antenatal care and skilled attendance at birth increased from less than 90% in 2000 to 93% in 2006, with small variations among states. Most importantly, there was a significant acceleration in the rate of decline of maternal mortality. Between 1990 and 2000, the indicator had dropped an average of 1.6% per year [35]. But the average rate of decline more than doubled between 2000 and 2006 to reach 2.7% per year, reflecting a drop in the number of maternal deaths from 72 to 58 per 100,000 live births, one of the lowest figures in Latin America [36,37]. It is important to stress that there was also a reduction of the gap between the rich states in the north and the poor ones in the south.

In the field of sexual and reproductive health, the MOH explicitly promoted the rights of women and carried out a number of innovative initiatives. One of the most relevant and by far the most controversial change was the revision of the national family planning policy through the introduction of three new contraceptive methods in the essential drug list: the sub-dermic implant, the female condom, and emergency contraception. The approval of the latter ignited an intense public debate, which provided useful lessons for Mexico and other countries dealing with similar challenges.

Emergency contraception was included in the essential drug list in July of 2005, after a lengthy and public discussion on its mechanisms of action, and associated risks and adverse effects, in which hundreds of organizations participated. The approval faced the strong opposition of the leaders of the Catholic Church, several conservative groups, and distinguished members of the party in power, all of whom argued, against nationally and internationally generated scientific evidence, that emergency contraception induces abortion [38]. Women’s rights advocates argued that access to emergency contraception was a top priority in a country with a high prevalence of unwanted pregnancies, an important proportion of which results from acts of sexual violence. Technical and scientific entities contributed to the debate by providing the scientific evidence about emergency contraception’s mechanisms of action and assessed public opinion, which was strongly in favor of the inclusion of this additional tool in the family planning guidelines and public services.

At the peak of the controversy, the office of the President of Mexico supported the inclusion of emergency contraception into the essential drug list stressing that it was the recommendation that resulted from a participatory and transparent process based on the analysis of scientific evidence. Such a strong endorsement was the positive culmination to a debate that in other Latin American countries has produced major political damage [39].

According to a review of opinion surveys, this was probably one of the most popular public policy measures adopted by that particular administration. In a predominantly Catholic nation, this decision was backed by the majority of women even within the most religious segments of the population. A survey implemented in 2004 to assess public opinion on emergency contraception in Mexico City showed an approval rate of 68.4% [40].

Three basic lessons can be drawn from the experience around the introduction of emergency contraception in the list of essential drugs of public institutions in Mexico: first, that scientific evidence can provide major support for controversial policy decisions; second, that it is important to take advantage of political opportunities and work collaboratively with strategic allies, such as civil society organizations; and third, that given the wave of democratization that is spreading in developing countries, we should not underestimate people’s aspiration for alternatives. In this particular case, Mexicans clearly rejected the interference of religious institutions in their private lives, especially in issues related to sexuality.

At the same time, the Government of Mexico adopted a gender and life-course perspective for its efforts to improve women’s health, identifying two critical priorities: gender-based violence and cancer. In 2003 the MOH implemented the first National Survey on Violence against Women, which showed that the prevalence of intimate partner violence among users of health services was 21.6%, *i.e.* one out of every five women had suffered from intimate partner violence in the 12 months prior to the survey [41].

Based on these findings and those of other studies, in 2006 the Mexican Congress passed a new law (*Ley General de Acceso de las Mujeres a una Vida Libre de Violencia*, in English, General Law Guaranteeing Access to All Women to a Life Free of Violence) that punishes psychological, physical, and patrimonial violence against women, and mandates the immediate arrest of the presumptive aggressor and the protection of the victim [42]. Women's organizations welcomed the law's passage, recognized it as an important step forward, and committed themselves to support its enforcement.

Cancer among women in low- and middle-income countries is another challenge that deserves more attention. There is evidence that shows that it has been a leading cause of death and disability worldwide for already many years [43]. But the distribution of cancer is changing and it is now increasingly affecting developing regions: 55% of the new cases of cancer currently occur in poorer nations and this figure could reach 60% by 2020 and 70% by 2050 [44,45].

The health transition described above encompasses the fundamental shift driving the rise of cancer in the developing world. Its protracted and complex nature is reflected in the coexistence of cervical and breast cancer [46]. Cervical cancer was dramatically reduced in developed countries even before its infectious nature was discovered and an effective vaccine was developed; therefore, it has been traditionally categorized as part of the “unfinished women’s health agenda”, along with infectious diseases, malnutrition and reproductive health problems. In contrast, breast cancer is an exemplar of the emerging challenges increasingly affecting developing countries. Cervical cancer, which has become a rare disease in rich nations, results in more than 200,000 deaths annually in developing countries. Contrary to common perception, breast tumors are now the number one cause of cancer-related deaths in women in all but the poorest nations of the world. Developing countries account for 46% of the one million new cases of breast cancer diagnosed each year worldwide and for 55% of the resulting deaths [47].

In Mexico mortality figures for cervical cancer have decreased consistently over the past two and a half decades due to increasing coverage of its early detection and treatment [48,49]. However, it still produces more than 4,000 deaths a year. Breast cancer mortality has doubled in the last 20 years, and it is now the second cause of death among women 30 to 54 years old [50].

Meeting these challenges required a two-pronged strategy involving both improved early detection and treatment. In terms of the former, the effective coverage of Pap smears increased from 36% to 41% between 2000 and 2006, while the coverage of mammography in women aged 40 to 69 years grew from 12% to 21% during the same period [17]. As part of the new insurance scheme created by the recent reform, a separate fund was established to finance the treatment of a package of catastrophic diseases, including cervical and breast cancer, the coverage of which is now universal. Nevertheless, closing the gaps in access to prevention and treatment among states remains a challenge.

While these public health efforts were put in place, cultural factors were also addressed. By and large, in the developed world cancer has been increasingly recognized as a disease that can be detected in its early stages and successfully treated. In contrast, in many developing countries, cancer continues to be hindered by the trappings of prejudice and stigma. Concerned about the possibility of being abandoned by their spouses when discovered ill, many women opt for not using available preventative services or, when a problem is detected, not to receive the proper treatment, mastectomy in particular [51,52]. For this reason, in Mexico the fight against cancer is being visualized also as a struggle against the social scourge of ignorance, stigma, discrimination, *machismo,* and the dehumanizing attempt to reduce women to a part of their bodies [53]. This topic has gained increasing visibility in the events commemorating the International Breast Cancer Day and has been openly addressed by the highest health authorities of the country and the President of Mexico himself [54].

Finally, the women and health aspect of the Mexican reform also embraced gender as a central issue in the health system. In 2003, the MOH established the National Center for Gender Equity and Reproductive Health [55]. This entity has the authority to suggest national policies related to sexual and reproductive health and monitor and evaluate these policies as well as the quality of public maternal and reproductive health services. It has also promoted the adoption of a cross-cutting gender perspective that has translated into gender-sensitive budgets, health information disaggregated by sex, and surveillance of gender biases in access to health services and quality of care. The Center has also started to address the role of women as informal providers of care for family members with chronic diseases; as traditional practitioners in the health teams serving indigenous communities; and as a growing component of the formal health workforce.

## Summary

The most important women and health challenges are far from over. There are still major threats that need to be addressed urgently. Salient among them are the further acceleration of the decline of maternal mortality to achieve MDG 5 and the attention to neglected emerging problems such as breast cancer and depression. Notwithstanding the importance of these problems, we should also recognize that several conceptual and empirical improvements in the field of women’s health have been recently achieved, with impacts both at the global and local levels. Salient among them are the global decline of maternal mortality figures, the innovative approaches to women’s health, and the successful implementation of comprehensive policies to address the comprehensive women and health agenda at the national level, as exemplified by the Mexican health reform experience. Future initiatives in this field should take advantage of this progress and build on it.

The Mexican experience shows that broad reform efforts can be used to design and implement specific initiatives addressing priority needs. In this particular case, these were interventions to improve the reproductive and sexual rights of girls and women.

This reform experience, due to its novel nature, also offered the opportunity to move beyond traditional approaches to women’s health to build a comprehensive agenda, which also includes neglected and emerging challenges such as gender-based violence, breast cancer, and the introduction of the gender perspective in the design, implementation, and evaluation of health policies.

Finally, we should stress that the successful implementation of the women and health approach in Mexico resulted from the establishment of creative alliances between the government and various important actors of the womenÂ´s health field, including researchers, women’s groups, other NGOs and the media.

The women and health approach represents an essential contribution to the advancement of the unfinished women’s health agenda both from a human rights and a development perspective, at the national and global levels.

## Competing interests

The first author (JF) was Minister of Health of Mexico during the period covered by this paper. The second author (OGD) was Director General for Performance Evaluation at the Ministry of Health of Mexico during the period covered by this paper. The third author (AL) was a frequent advisor to the Ministry of Health of Mexico on women’s health issues during the period covered by this paper.

## Authors’ contributions

The three authors participated equally in the development of the outline for this paper, in the gathering of all relevant literature and information, and in the actual writing of the essay. All authors read and approved the final manuscript.

## Pre-publication history

The pre-publication history for this paper can be accessed here:

http://www.biomedcentral.com/1472-6874/12/42/prepub
